# Point-of-care NT-proBNP monitoring for heart failure: observational feasibility study in primary care

**DOI:** 10.3399/BJGPO.2022.0005

**Published:** 2022-07-13

**Authors:** Jason Chami, Susannah Fleming, Clare J Taylor, Clare R Bankhead, Tim James, Brian Shine, Julie McLellan, FD Richard Hobbs, Rafael Perera

**Affiliations:** 1 Sydney Medical School, University of Sydney, Sydney, Australia; 2 Nuffield Department of Primary Care Health Sciences, University of Oxford, Oxford, UK; 3 Department of Clinical Biochemistry, Oxford University Hospitals NHS Foundation Trust, Oxford, UK

**Keywords:** brain natriuretic peptide, heart failure, point-of-care testing, primary health care, family practice, general practice

## Abstract

**Background:**

Around one million individuals in the UK have heart failure (HF), a chronic disease that causes significant morbidity and mortality. N-terminal pro-B-type natriuretic peptide (NT-proBNP) monitoring could help improve the care of patients with HF in the community.

**Aim:**

The aim of this study is to provide evidence to support the routine use of point-of-care (POC) NT-proBNP monitoring in primary care.

**Design & setting:**

In this observational cohort study, the Roche Cobas h 232 POC device was used to measure NT-proBNP in 27 patients with HF at 0, 6, and 12 months, with a subset reanalysed in the laboratory for comparison.

**Method:**

Data were analysed for within-person and between-person variability and concordance with laboratory readings using Passing–Bablok regression. GPs reported whether POC results impacted clinical decisionmaking, and patients indicated their willingness to participate in long-term cohort studies using the Likert acceptability scale.

**Results:**

Within-person variability in POC NT-proBNP over 12 months was 881 pg/mL (95% confidence interval [CI] = 380 to 1382 pg/mL). Between-person variability was 1972 pg/mL (95% CI = 1,525 to 2791 pg/mL). Passing–Bablok regression showed no significant systematic difference between POC and laboratory measurements. Patients indicated a high level of acceptability, and GP decisionmaking was affected for at least one visit in a third of patients.

**Conclusion:**

Within-person variability in POC NT-proBNP is around half of between-person variability, so detecting changes could be of use in HF management. High patient acceptability and impact on clinical decisionmaking warrant further investigation in a larger long-term cohort study.

## How this fits in

NT-proBNP is used to diagnose and manage HF in the UK. POC NT-proBNP assays currently lack evidence for incorporation into routine clinical practice in primary care. This study showed that POC NT-proBNP testing is acceptable to patients, feasible for implementation in UK general practice, and sufficiently accurate. Most importantly, GPs were able to use this new technology to improve the care of more than one-third of patients with HF in primary care.

## Introduction

Every year in the UK, more than 190 000 people are newly diagnosed with HF,^1^ a chronic disease that carries significant risk of morbidity and mortality. As the disease burden continues to grow, it is important to optimise routine care for patients in the community.

In the UK, natriuretic peptide (NP), a collective term for B-type natriuretic peptide (BNP) and N terminal pro-B type natriuretic peptide (NT-proBNP), levels are currently used as part of the diagnostic pathway for HF and have been shown to correlate with poor prognosis.^2^ Patients with established HF receive much of their care in the community, either in primary care or through specialist community HF nurses. These patients may only be referred back to secondary or tertiary care if they experience acute illness. There is a clinical need for early identification of HF deterioration, before patients require hospital referral or admission.

It has therefore been postulated that the routine monitoring of NP in a UK community setting could assist in improving care of patients with HF.^3^ However, given the delays associated with the return of laboratory measurements, and the National Institute for Health and Care Excellence guidance recommendation for use only as part of treatment optimisation within specialist care,^2^ NP testing is not routinely used as part of HF management in UK primary care. Recent advances have made POC NP testing possible,^4^ but there is currently limited evidence to support the use of such devices as part of routine HF monitoring in UK primary care. Both BNP and NT-proBNP (NT-proBNP is the inactive part of the hormone proBNP, secreted by cardiomyocytes due to wall stress) appear to have similar predictive value in HF,^3,5^ but NT-proBNP has less biological variation than BNP and greater stability when stored at room temperature in EDTA plasma.^4,6–11^ Unlike BNP assays, many NT-proBNP assays use the same antibodies, so results from different manufacturers show greater agreement.^12^ For these reasons, NT-proBNP is an attractive target for routine monitoring of HF in the community.

The best clinical biomarkers give reliable results upon repeat testing and capture true differences in disease progression between patients; in other words, low within-person variability when compared to between-person variability. The principal aim of this study was therefore to determine the within-person and between-person variability in POC NT-proBNP measurements in patients with HF in primary care settings. This study also aimed to assess the feasibility of POC NT-proBNP monitoring for HF in primary care.

## Method

A previous horizon scan of POC NP devices suitable for use in the diagnosis of HF in primary care identified two potential devices.^5^ Of these, the authors chose to use the Roche Cobas h 232 device (Roche Diagnostics, Switzerland), as this was both the device for which the most evidence was available and the only one for which a UK distributor could be identified. Furthermore, it carries a CE mark and is currently marketed in the UK for use in the diagnosis of HF.

This study recruited patients from three GP practices in Oxfordshire. Prospective patients were identified by searching the practice register for adults with a recorded diagnosis of HF. These were checked by a clinician against the inclusion and exclusion criteria for the study (Table 1). Eligible patients were invited to take part by post or direct invitation.

**Table 1.** Study inclusion and exclusion criteria

		Summary *n* (%)
**Mean age, years (standard deviation)**		77.6 (9.1)
**Sex**	Male	10 (37.0)
	Female	17 (63.0)
**Ethnicity**	White British	25 (92.6)
	White Other	1 (3.7)
	Indian	1 (3.7)
**Type of heart failure**	Heart failure with preserved ejection fraction	10 (37.0)
	Heart failure with reduced ejection fraction	13 (48.1)
	Unknown	4 (14.8)
	Class I	8 (29.6)
	Class II	14 (51.9)
	Class III	4 (14.8)
	Class IV	1 (3.7)
**Comorbidities**	Hypertension	15 (55.5)
	Chronic kidney disease	10 (37.0)
	Arthritis	9 (33.3)
	Cancer	6 (22.2)
	Stroke	5 (18.5)
	Atrial fibrillation	5 (18.5)
	Asthma	5 (18.5)

Participants attended three scheduled visits at 0, 6, and 12 months from baseline. Visits were at the participant’s usual GP practice, with a practice nurse trained in the use of the POC device. At each of the three visits, three venous blood samples were taken: one for POC NT-proBNP measurement, and two to be sent to the local laboratory for NT-proBNP and renal function testing.

The POC Roche Cobas h 232 instrument requires 150 µL of heparinised blood to be prepared on a Roche Cardiac proBNP test strip (Roche Diagnostics, Switzerland). It has an analytical range of 60–9000 pg/mL and takes 12 minutes to produce a result.^13^ The laboratory samples for NT-proBNP were tested using the Abbott Architect i2000 immunoassay analyser (Abbott Diagnostics, Maidenhead, UK).

This study also requested that where possible, additional POC NT-proBNP measurements should be made when study participants attended the practice outside of their scheduled visits for any reason related to their HF. Following each appointment, each patient’s clinician was provided with available results (including any laboratory results that had returned at that point) and was asked whether the NT-proBNP measurement would change their patient management.

Finally, the authors assessed the acceptability of the POC test using Likert scores, scored by patients at the final study visit.

## Statistics and sample size

As this is a feasibility study, the sample size of 30 patients was chosen based on practical judgement rather than a formal calculation.

Where results from the POC device were reported below or above the analytical range of the instrument, this study analysed them using the value closest to the reporting limit (values reported as <60 pg/mL were analysed as 59 pg/mL and those reported as >9000 pg/mL were analysed as 9001 pg/mL). In 5 cases, POC NT-proBNP levels were mistakenly entered as laboratory NT-proBNP; these data were removed.

For each person, the study calculated the mean (within-person mean) and standard deviation (within-person standard deviation) of their measurements. The authors calculated within-person variability by taking the mean of the individual within-person standard deviations. The between-person variability was calculated by taking the standard deviation of the individual within-person means.

Analysis was performed using R (version 4.0.3). The coefficient of variation (CV) of within-patient POC readings was plotted to estimate precision. Furthermore, Passing-Bablok regression,^13^ a Bland–Altman plot,^14^ and mean difference calculations were used to compare POC NT-proBNP readings to matched laboratory-measured NT-proBNP readings. Unfortunately, 48 out of 72 NT-proBNP blood samples from the study were either not processed or not prepared appropriately, since the primary HF marker under analysis at the laboratory at the time was BNP, not NT-proBNP. As this error occurred throughout the study period and was not related to a single event it was concluded that missing data occurred in a random manner, so complete case analysis was used. The authors also confirmed that missingness was not associated with a patient’s pulse, blood pressure, weight, POC NT-proBNP, eGFR, or serum creatinine.

## Results

### Descriptive statistics of the population

**Figure 1.**
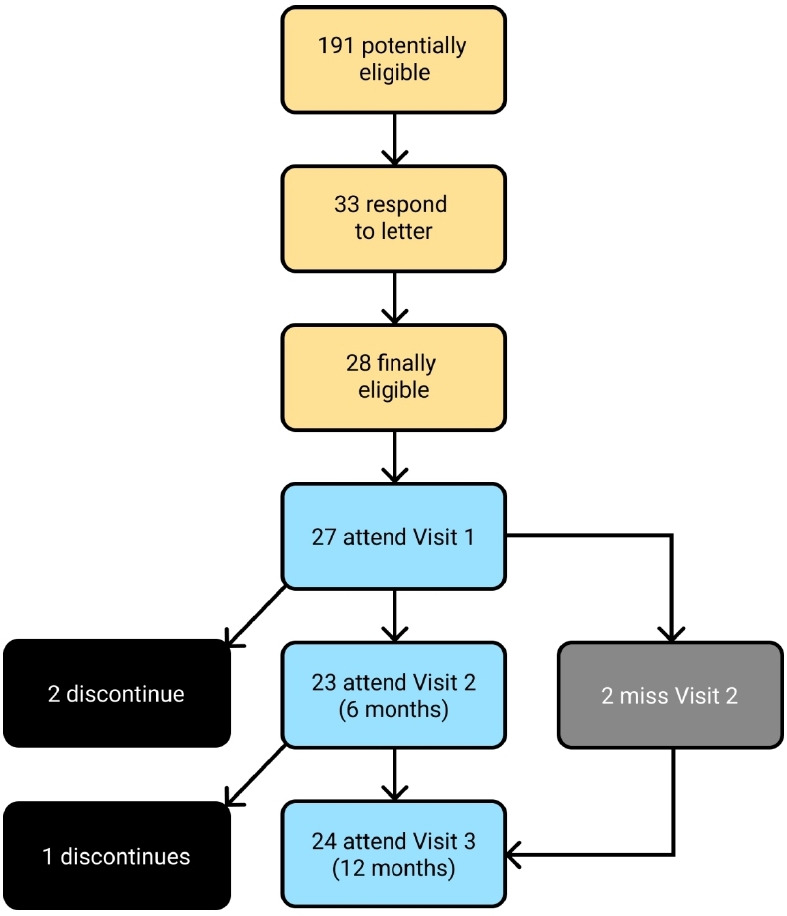
Flow of participants in the observational study of point-of-care N-terminal pro-B-type natriuretic peptide. Patients noted as discontinued did not attend any further visits. The two participants who missed Visit 2 but were not discontinued continued to attend at Visit 3.

Recruitment to the study took place from 20 February to 28 March 2018, with follow up to 29 March 2019. The study recruited 27 participants from three GP practices to take part in the study. Figure 1 shows participant flow through the study. The median follow-up time was 366 days, with a maximum of 396 days, and the total study follow-up time was 24.5 person-years. Three participants discontinued participation during the study: two of these only attended Visit 1, and one participant attended both Visit 1 and Visit 2. Two participants discontinued due to changes in circumstances, and one participant died during the course of the study.

A total of 191 letters were sent to potentially eligible patients from three GP practices. Of these, 33 (17%) were returned, of which 28 were finally deemed eligible. All but one of those eligible consented to participate in the study. Overall, 27 patients were recruited, yielding a recruitment rate of 14%.

Two participants missed Visit 2 during the study but were not discontinued as they attended Visit 3. Therefore, 91% of planned visits were performed, and POC NT-proBNP measurements were successfully obtained at all visits attended by participants. No patient in the study had POC NT-proBNP measured at a routine GP appointment. Table 2 summarises the baseline characteristics of the study population.

**Table 2.** Baseline characteristics of study participants. Comorbidities reported in less than five participants have not been presented

Inclusion criteria	Exclusion criteria
Willing and able to give informed consent Male or female Aged 18 or above Confirmed diagnosis of heart failure made by cardiologist and/or echocardiography Currently managed in primary care Clinician willing to offer routine POC NT-proBNP monitoring	Terminally ill or receiving palliative care for a condition other than heart failure at time of recruitment

NT-proBNP = N-terminal pro-B-type natriuretic peptide. POC = Point-of-care.

### Primary analysis

Within-person variability in POC NT-proBNP over 12 months (*n* = 25) was 881 pg/mL (95% CI = 380 to 1382 pg/mL). Between-person variability in POC NT-proBNP over 12 months (*n* = 27) was 1972 pg/mL (95% CI = 1525 to 2791 pg/mL).

### Secondary analyses

#### Device and operator performance

Extant laboratory measurements (*n* = 24) covered a concentration range of 101.7–10 703.4 pg/mL. There were no significant differences in any clinical or laboratory values, including POC NT-proBNP in patients with or without laboratory NT-proBNP measurements.

**Figure 2.**
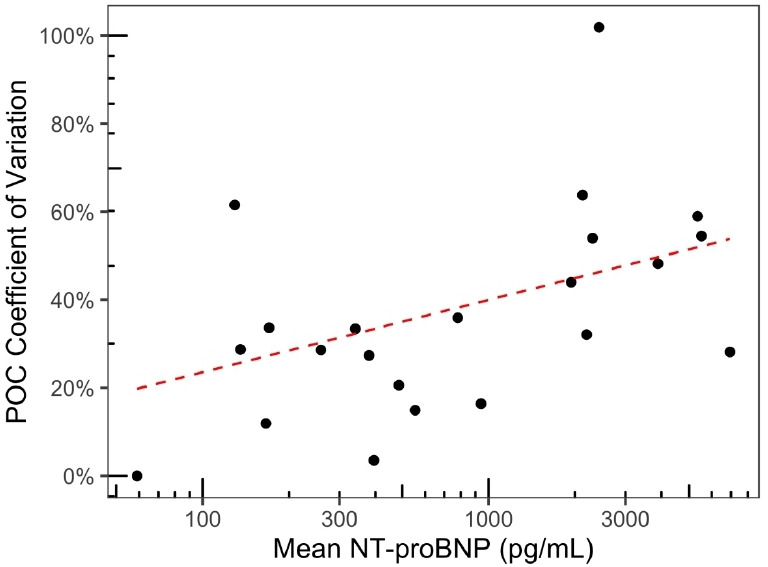
Coefficient of variation (CV) is calculated for the set of point-of-care (POC) N-terminal pro-B-type natriuretic peptide (NT-proBNP) readings for each patient, plotted against the mean POC NT-proBNP reading for each patient. CV increases with the mean NT-proBNP reading.

Plotting the CV of within-patient POC readings against the mean of within-patient POC readings showed that within-patient variation increased with mean NT-proBNP level (Figure 2). The CV across the full range of acquired samples was 37.6% (95% CI = 32.0–43.1%).

**Figure 3.**
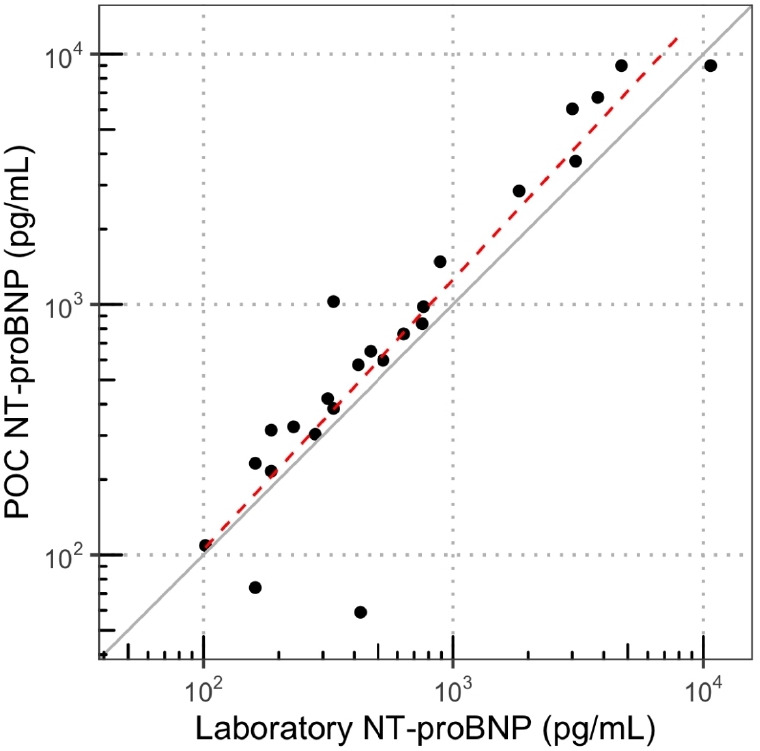
Scatter plot and Passing and Bablock regression of log-transformed point-of-care (POC) and laboratory N-terminal B-type natriuretic peptide (NT-proBNP) showing close correlation (R^2^ = 0.9). Intercept: –0.3 (95% confidence interval (CI) = –1.6–1.0). Slope: 1.1 (95% CI = 0.9–1.3).

**Figure 4.**
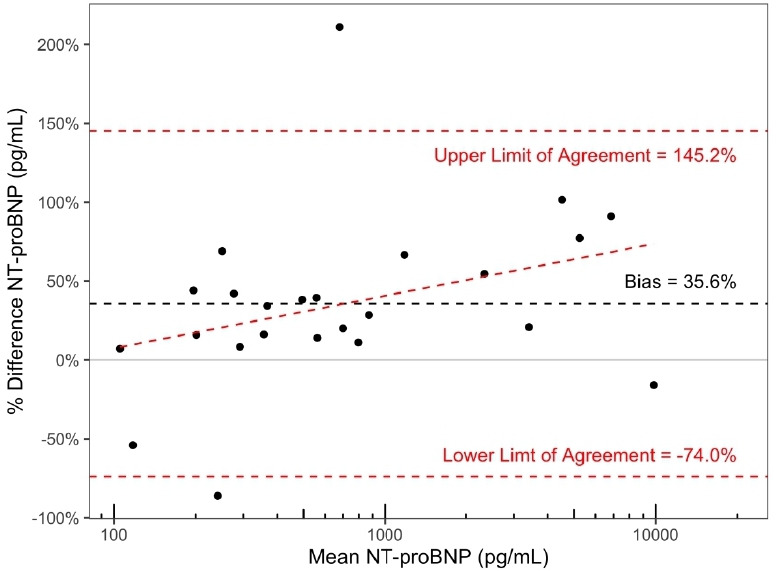
Bland–Altman plot of point -of-care (POC) and laboratory N-terminal B-type natriuretic peptide (NT-proBNP), with the mean of the laboratory and POC readings on the *x*-axis, and the percentage difference on the *y*-axis (calculated as POC – Laboratory / Laboratory). POC readings are 35.6% higher on average than paired laboratory readings (95% confidence interval (CI) = 12.0 to 59.2%). Upper and lower limits of agreement are 145.2% and –74.0% respectively.

A Passing–Bablok regression^13^ was performed on the extant data (Figure 3), comparing POC NT-proBNP readings to the laboratory measurement. The data were log-transformed since the differences between the POC and laboratory NT-proBNP readings were dependent on the magnitude of the reading itself. The slope was estimated at 1.1 (95% CI = 1.0 to 1.2), and the intercept was estimated at –0.46 (95% CI = -1.6 to 0.5) suggesting no significant systematic difference in measurements. On the other hand, a Bland–Altman plot on POC readings as a percentage of matching laboratory readings, with mean NT-proBNP displayed on a log-scale (Figure 4) revealed that POC NT-proBNP levels were significantly higher than laboratory readings (mean 35.6%; 95% CI = 12.0 to 59.2%). Upper and lower limits of agreement were 145.2% and -74.0% respectively. Fitting a linear model on the Bland–Altman plot with mean NT-proBNP log-transformed as in Figure 4 yielded an intercept of -59.0% (95% CI = -178.7 to 60.7%) and a slope of 0.1 (95% CI = -0.03 to 0.3), neither of which was significantly different from zero.

#### Impact on decision-making

Overall, 19% of POC NT-proBNP tests caused a change in the decisionmaking process. This means that the decisionmaking process was affected for at least one visit in 37% of patients. All doctors reported that their decision to change treatment would be the same if only the POC result were available, without the laboratory NT-proBNP result. In 12% of the decisions not to change treatment, doctors said their decision may have been different if they had access to laboratory NT-proBNP data instead of the POC result.

#### Willingness to participate in long-term cohort studies

Of the 24 individuals who reached the end of the study, 19 individuals (79%) rated POC NP measurement as ’Totally Acceptable‘, 4 individuals (17%) as ’Acceptable‘, and one individual (4%) as ’Neutral‘. Only two individuals discontinued willingly, citing personal circumstances. One patient died during the course of the study. Therefore, 24 patients completed the study, giving a retention rate of 89% of total patients, or 94% of surviving patients.

## Discussion

### Summary

This study found that it was feasible to do routine POC NT-proBNP testing in primary care, with testing successfully carried out at every planned study visit. GPs used the results of the POC tests to inform changes to more than a third of treatment plans, and patients found POC testing acceptable. Between-person variability in POC NT-proBNP was around twice as large as within-person variability over a 12-month period.

### Comparison with existing literature

The CV for within-patient POC readings over the course of the year was relatively low at 37.6%, and lowest at reduced concentrations where a diagnosis of HF is generally least obvious.^15^ Since each patient’s readings were taken 6 months apart, this CV reflects instrument variation as well as biological variation over the year-long study.

Comparison to laboratory NT-proBNP results showed good agreement, with the POC device showing slight positive bias, as has been previously reported by the Scandinavian Evaluation of Laboratory Equipment for Primary Health Care, and Hex *et al*.^16,17^ Although simple bias calculations revealed a significant positive bias, non-significant results from the Passing–Bablok regression and linear regression on the Bland–Altman plot suggest that this bias is only borderline significant and is acceptably small for a primary care setting. Moreover, the magnitude of difference between the POC and laboratory devices is similar to those seen between the various laboratory assays for BNP and NT-proBNP produced by different manufacturers,^18,19^ as immunoassays vary with respect to antibody specificity^20^ and the population characteristics of those assessed.^21^ Perhaps more important are the few recorded cases where there has been significant variation from visit to visit, and lack of concordance between POC and laboratory readings (Figures 2 3), suggesting operator error rather than measurement bias. If POC testing is to be incorporated into routine primary care, it is imperative that operators are trained in its usage, and checks are developed to prevent and identify irregular results.

^15^

### Strengths and limitations

This study had some limitations. Firstly, the sample size was limited due to a low response rate from invited patients. Despite this, the study was able to draw statistically significant conclusions and clearly determine both that a larger cohort study of POC NT-proBNP testing is feasible, and that it is possible to routinely carry out planned POC NT-proBNP testing in primary care. Another limitation was the accidental loss of two-thirds of planned laboratory NT-proBNP tests due to a laboratory error. This has limited the robustness of the data comparing POC and laboratory results. Nonetheless, a comparative analysis was still possible using the remaining data, and results were comparable to previous research.^17^ Finally, POC testing only occurred at planned appointments during the study, and not at any unplanned appointment. This likely reflects current pressures in UK primary care. If unscheduled NT-proBNP testing is to be successfully used, other factors such as sufficient time, training, and staff available to carry out testing will need to be considered. Nonetheless, this study's data indicate that future research into the importance of operator skill, lack of trust in POC devices, and short-term variability of measurements, is all feasible. As this was a feasibility study, the authors did not follow patients up to identify hospital admission following POC testing, but future research could investigate the ability of primary care POC testing in HF to avoid hospital admissions or readmission.

The unique strength of this work was its pragmatic design. Only one or two nurses per practice were trained on the new device, the population was typical of those who would benefit from close monitoring in primary care, and the device used is available to GPs in the UK today.

### Implications for practice

More than a third of patient treatment plans were altered because of the POC NT-proBNP readings, suggesting that clinicians found the added information useful. Moreover, in all cases of altered treatment, doctors noted that their decision would have been the same regardless of their access to confirmatory laboratory findings. Adlbrecht *et al* found that the Roche Cobas h 232 POC NT-proBNP measurement device had a 100% negative predictive value for HF-related hospitalisation, and better positive predictive value than any clinical sign or symptom when NT-proBNP was in the low range of 100–500pg/mL.^15^ In precisely these low concentrations, this study has shown that POC testing has the greatest precision. Therefore, for patients on the borderline of current diagnostic cut-offs who may never be referred to a hospital for BNP or NT-proBNP testing may stand to benefit greatly from POC testing in primary care. Moreover, POC testing is cheaper and more accessible in terms of instrument costs, time, and personnel, making implementation in primary care cheaper than laboratory testing. On the other hand, in 12% of the decisions not to change treatment, doctors said their decision may have been different if they had access to laboratory data instead of the POC device, suggesting a potential lack of trust in the POC device.

In this setting, NT-proBNP monitoring was found to be acceptable to patients, feasible for implementation in UK general practice, and sufficiently accurate, especially for the patients on the border of clinical cut-offs who may stand the most to gain. Most importantly, GPs were willing to use this new technology to improve the care of more than one-third of patients with HF in primary care.

## Data

The data underlying this research is not appropriate for open publication. However, it can be accessed by other researchers on application to the authors.

## References

[1] Conrad N, Judge A, Tran J (2018). Temporal trends and patterns in heart failure incidence: a population-based study of 4 million individuals. Lancet.

[2] National Institute for Health and Care Excellence (2018). Chronic heart failure in adults:diagnosis and management [NG106]. www.nice.org.uk/guidance/ng106.

[3] National Clinical Guideline Centre (UK) (2010). Chronic Heart Failure: National Clinical Guideline for Diagnosis and Management in Primary and Secondary Care: Partial Update [NICE Clinical Guidelines, No. 108].

[4] Yeo KTJ, Wu AHB, Apple FS (2003). Multicenter evaluation of the Roche NT-proBNP assay and comparison to the Biosite Triage BNP assay. Clin Chim Acta.

[5] Denny N, Lasserson D, Price CP (2011). Diagnostic technology: point of care B-type natriuretic peptide testing.

[6] Bruins S, Fokkema MR, Römer JWP (2004). High intraindividual variation of B-type natriuretic peptide (BNP) and amino-terminal proBNP in patients with stable chronic heart failure. Clin Chem.

[7] Frankenstein L, Remppis A, Frankenstein J (2009). Variability of N-terminal probrain natriuretic peptide in stable chronic heart failure and its relation to changes in clinical variables. Clin Chem.

[8] Meijers WC, van der Velde AR, Muller Kobold AC (2017). Variability of biomarkers in patients with chronic heart failure and healthy controls. Eur J Heart Fail.

[9] Nordenskjöld AM, Ahlström H, Eggers KM (2013). Short- and long-term individual variation in NT-proBNP levels in patients with stable coronary artery disease. Clin Chim Acta.

[10] Schindler EI, Szymanski JJ, Hock KG (2016). Short- and long-term biologic variability of galectin-3 and other cardiac biomarkers in patients with stable heart failure and healthy adults. Clin Chem.

[11] Takeda Y, Takeda Y, Suzuki S, Kimura G (2009). Within-person variation of the plasma concentration of B-type natriuretic peptide: safety range in stable patients with heart failure. Am Heart J.

[12] Semenov AG, Katrukha AG (2016). Analytical issues with natriuretic peptides - has this been overly simplified. EJIFCC.

[13] Bablok W, Passing H (1985). Application of statistical procedures in analytical instrument testing. J Automat Chem.

[14] Altman DG, Bland JM (1983). Measurement in medicine: the analysis of method comparison studies. Statistician.

[15] Adlbrecht C, Neuhold S, Hülsmann M (2012). NT-proBNP as a means of triage for the risk of hospitalisation in primary care. Eur J Prev Cardiol.

[16] Jacobsen CE (2013). Report from the evaluation SKUP/2013/97 of NT-proBNP on Cobas h 232.

[17] Hex C, Smeets M, Penders J (2018). Accuracy, user-friendliness and usefulness of the Cobas h232 point-of-care test for NT-proBNP in primary care. J Clin Pathol.

[18] Clerico A, Zaninotto M, Prontera C (2012). State of the art of BNP and NT-proBNP immunoassays: the CardioOrmoCheck study. Clin Chim Acta.

[19] Mueller T, Gegenhuber A, Poelz W, Haltmayer M (2003). Comparison of the Biomedica NT-proBNP enzyme immunoassay and the Roche NT-proBNP chemiluminescence immunoassay: implications for the prediction of symptomatic and aymptomatic structural heart disease. Clin Chem.

[20] Saenger AK, Rodriguez-Fraga O, Ler R (2017). Specificity of B-type natriuretic peptide assays: cross-reactivity with Ddfferent BNP, NT-proBNP, and proBNP peptides. Clin Chem.

[21] Dai Z, Asano T, Takahashi O (2020). The minimal informative monitoring interval of N-terminal pro-B-type natriuretic peptide in patients with stable heart failure. BMC Cardiovasc Disord.

